# Sudden infant death syndrome due to long QT syndrome: a brief review of the genetic substrate and prevalence

**DOI:** 10.1186/s40709-017-0063-1

**Published:** 2017-03-14

**Authors:** Nikolaos S. Ioakeimidis, Theodora Papamitsou, Soultana Meditskou, Zafiroula Iakovidou-Kritsi

**Affiliations:** 1 Laboratory of Histology and Embryology, Faculty of Medicine, Aristotle University of Thessaloniki, Aristotle University of Thessaloniki Campus, 54124 Thessaloníki, Greece; 2 Laboratory of Medical Biology-Genetics, Faculty of Medicine, Aristotle University of Thessaloniki, Aristotle University of Thessaloniki Campus, 54124 Thessaloníki, Greece

**Keywords:** Sudden infant death, Long QT syndrome, Prevalence

## Abstract

The pathophysiological mechanisms which lead to sudden infant death syndrome (SIDS) are not completely understood. Cardiac channelopathies are a well-established causative factor with long QT syndrome (LQTS) being the most frequent one, accounting for approximately 12% of SIDS cases. The genetic substrate of the above arrhythmogenic syndrome has been thoroughly described but only specific gene mutations or polymorphisms have been identified as SIDS causative. The review will focus on the prevalence of LQTS-induced SIDS or near-SIDS cases and the mutations held responsible. A literature search was performed in PubMed and Scopus electronic databases. Search terms used were: long QT syndrome, channelopathies, QT prolongation, cardiac ion channels. The above-mentioned search terms were always combined with the term: sudden infant death syndrome. Study types considered eligible were: case–control, family pedigree analysis, case reports. The prevalence of LQTS-induced SIDS according to six broad genetic studies ranges from 3.9 to 20.6%, with an average of 12%. Since LQTS can be effectively managed, LQTS-related SIDS cases could be prevented, provided that a screening method is efficient enough to detect all the affected infants.

## Background

Cardiac ion channelopathies have been found to be implicated as a causative factor of sudden infant death syndrome (SIDS) by numerous studies. The first channelopathy hypothesized to be linked with SIDS was the long QT syndrome (LQTS) in a study conducted in 1976 by Schwarz [1]. Since then, Brugada Syndrome (BrS) and catecholaminergic polymorphic ventricular tachycardia (CPVT) have been listed as possible causes, with short QT syndrome (SQTS) the most recent addition to the list. It is remarkable that long QT syndrome has recently been found to account for almost 12% of SIDS cases [2]. The purpose of this review is to summarize and present major studies (mostly case–control) which investigate the relationship between the two pathological entities up to date. The review will focus on the prevalence of LQTS-induced SIDS or near-SIDS cases and the mutations held responsible. Since LQTS can be adequately managed, LQTS-related SIDS cases could be prevented, provided that a screening method is efficient enough to detect all the affected infants. The systematic classification of the detected gene variations could serve as a basis for discriminating the most common of them and thus facilitate the development of targeted genetic screening in order to prevent the tragic event of sudden death in infancy.

### Sudden infant death syndrome (SIDS)

Sudden infant death syndrome was first defined as a pathological entity in 1969 as “the sudden death of any infant or young child, which is unexpected by history, and in which a thorough post-mortem examination fails to demonstrate an adequate cause of death” [3]. However, the definition above was deemed too non-specific since it lacked an age limitation and was based solely on the post-mortem body dissection excluding the autopsy of the surrounding environment. This is the reason why in 1989 the National Institute of Child Health and Human Development (NICHD) set out to redefine SIDS [4]. The revised definition limited the age of the infant to under 1 year old (<1 year) and stated that the diagnosis of SIDS should only be made if a complete autopsy of the death scene and a clinical history (in addition to an unremarkable post-mortem examination) fail to identify any other specific cause of death [5]. In 2003, after years of controversies, Bruce Beckwith, the visionary of a solid definition, pointed out the need of re-evaluating the definition and categorizing SIDS cases so an expert panel was formed in 2004 in San Diego to address the matter [3, 6]. The San Diego definition as named, is therefore the most recent and systematic one and is widely accepted for the classification of SIDS cases [6]. It divides SIDS cases into three categories, according to specific clinical findings and autopsy results and modifies the 1989 definition by adding the condition that the death must occur during sleep.

Several factors have been accused of triggering SIDS such as maternal age, maternal race, alcohol and tobacco use during pregnancy, low birth weight, male gender, bed sharing and prone sleeping position [7–10]. Despite a notable decline of about 50% in SIDS related deaths, SIDS continues to be the leading cause of post-neonatal death in the USA even after the launch of the “Back to Sleep” campaign [11].

An accurate pathophysiological model able to explain SIDS is not yet established. Various “triple risk models” have been proposed, correlating environmental and intrinsic risk factors which subsequently lead to a vulnerable infant [12]. Injuries and neurochemical abnormalities of the brainstem have been linked to SIDS. Brainstem gliosis possibly causing hypoxemic events in infant victims was found to be related with maternal smoking [13]. Serotonin transporter gene polymorphisms undermining the infantile brainstem’s response to hypoxia or hypercapnia are also characterized as SIDS risk factors [14, 15]. Bacterial and viral infections as well as immunologic polymorphisms (interleukin-10 over-secretion, interleukin-1β and Vascular Endothelial Growth Factor overexpression) are considered triggering factors [16]. Infants with alterations of the intestinal flora, more specifically infant hosts of enterotoxin producing *Staphylococcus aureus*, are found to be more susceptible to SIDS than non-toxigenic *S. aureus* hosts [17]. Finally, arrhythmogenic cardiac ion channelopathies (LQTS, SQTS, BrS and CPVT) are established SIDS causative factors with LQTS being the most frequent one.

## Methods

A literature search was performed in PubMed (https://www.ncbi.nlm.nih.gov/pubmed) and Scopus (https://www.scopus.com/home.uri) electronic databases. The latest data retrieval date was December 2015. Search terms used were: long QT syndrome, channelopathies, QT prolongation, cardiac ion channels. The above-mentioned search terms were always combined with the term: sudden infant death syndrome. Abbreviations of the above syndromes were also used in the search. Studies had to satisfy the new definition of SIDS as well as the classification criteria, all of which were introduced in San Diego in 2004. Articles studying a sudden unexplained death population which contained a sub-population of SIDS cases were also included in the review. Study types considered eligible were: case–control, family pedigree analysis, case reports.

## Results

We identified 13 case–control studies investigating the prevalence of LQTS-induced SIDS [2, 18–29]. Six of them were broad genetic studies of an unselected SIDS population and screened cases for a majority of the LQTS-causing genes known at the time of the study [18–23], whereas the rest concentrated on one or two specific genes [24–29]. The prevalence of LQTS-induced SIDS according to the six broad studies ranged from 3.9 to 20.6%, with an average of 12% [18–23]. A separate and more detailed discussion of the results, regarding the implication of LQTS in SIDS, is provided right below.

## The role of long QT syndrome in sudden infant death syndrome

**Fig. 1:**
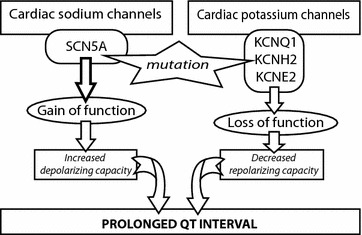
Functional effect of cardiac ion channel mutations on the repolarization/depolarization capacity of the myocardium

From a historical perspective, in February 1976, Schwartz published the completely novel hypothesis suggesting that LQTS could contribute to a number of SIDS cases [1]. In September of the same year, Maron et al. [30] proposed the connection between LQTS and SIDS. They studied 42 sets of parents who had at least one child with SIDS. In 1998, Schwartz et al. [31] confirmed their initial hypothesis through the results of their study which included 34,442 newborns and lasted for 18 years. They concluded that an evident prolongation of the QT interval on days 3 and 4 of life was associated with a higher risk of SIDS occurrence. Two years later, the first molecular evidence of the appearance of long QT syndrome in SIDS was published [32]. Since then, mutations in 7 genes, KCNQ1, KCNH2/HERG, SCN5A, KCNE2, CAV3, SCN4B, SNTA1 causing the LQTS, have been associated with SIDS cases [2, 18–29]. A brief schematic of the mechanism through which cardiac ion channel mutations lead to the prolongation of the QT interval is provided in Fig. 1.

In our review, we divided eligible studies in two separate subsets. The first one consists of broad genetic studies of an unselected SIDS population. Their results are summarized in Table 1. These studies performed mutational analysis of all the LQTS-related genes known at the time they were conducted. The second subset consists of studies investigating the prevalence of SIDS-causative mutations of one specific gene or two, and is presented in Table 2.

**Table 1:** Long QT syndrome as a causative factor of SIDS and implicated mutations—Broad genetic studies

Study/year	Study type	Total number of cases	Number of SIDS cases with causative mutation	Genes	Mutations
Wang et al./2014 [18]	Case–control	141	19 (13.4%)	SCN5A	p.L618F, p.P1011L*, p.A1100V, p.T1131I, p.T1304M, p.M1487L, p.Q1832E, p.R1944X*, p.D501G, p.A662S*, p.A997D*, p.A1106S*, p.S1285G*, p.A149V*, p.E1890K*
				KCNQ1	p.Y51C*, p.P99R*, p.N593K*
				KCNH2	p.D982N*
				KCNE2	p.M54T
Glengarry et al./2014 [19]	Case–control	102	4 (3.9%)	SCN5A	p.I759F, p.F1522Y*
				KCNH2	p.R1047L
				KCNQ1	p.E146K
Horigome et al./2010 [20]	Case–control	58	12 near SIDS (20.6%)	SCN5A	p.V176M, p.N406K, p.R1623Q, p.L1772V
				KCNH2	p.A561V, p.G628S, p.T613M, p.G572S, p.A614V, p.N633S
				KCNQ1	p.G643S, p.G269S
Millat et al./2009 [21]	Case–control	32	5 (15.6%)	SCN5A	p.R975W, p.Q692K, p.S1333Y
				KCNH2	p.R148W
				KCNQ1	p.G626S
				KCNE1	p.T20I
Otagiri et al./2008 [22]	Case–control	42	4 (9.5%)	SCN5A	p.G1084S, p.F1705S, p.F532C
				KCNH2	p.T895M
				KCNQ1	p.K598R
Arnestad et al./2007 [2]	Case–control	201	19 (9.4%)	SCN5A	p.S216L, p.A586_L587del, p.R680H, p.T1304M, p.F1486L, p.V1951L, p.F2004L, p.P2006A
				KCNH2	p.R273Q, p.R954C, p.K897T
				KCNQ1	p.G460S
				KCNE2	p.Q9E
				CAV3	p.T78M

* Sequence variation is previously unreported and is of the type which may or may not be causative of the disorder

**Table 2:** Long QT syndrome as a causative factor of SIDS and implicated mutations—Genetic studies focusing on one single or two genes

Study/year	Study type	Total number of cases	Number of SIDS cases with causative mutation	Genes	Mutations
Kato et al./2014 [23]	Case–control	7	4	SCN5A	p.N1774D, p.F1486del, p.N406K
				KCNH2	p.G628D
Tan et al./2010 [24]	Case–control	292	1 (0.3%)	SCN4B	p.S206L*
Cheng et al./2009 [25]	Case–control	292	3 (1%)	SNTA1	p.S287R, p.T372M, p.G460S
Cronk et al./2007 [26]	Case–control	134	3 (2.2%)	CAV3	p.V14L, p.T78M, p.L79R
Plant et al./2006 [27]	Case–control	133	7 (5.2%)	SCN5A	p.S1103Y, p.S524Y, p.R689H, p.E1107K
Lupoglazoff et al./2004 [28]	Case–control	23	3 (13%)	KCNH2	p.G604S
Ackerman et al./2001 [29]	Case–control	93	2 (2.1%)	SCN5A	p.A997S, p.R1826H

* Sequence variation is previously unreported and is of the type which may or may not be causative of the disorder

It is obvious that the most accurate way to estimate the prevalence of LQTS-induced SIDS is to summarize the results of major genetic studies. Those, include a sufficient enough population to reach safe conclusions, have an extended data collection period and most importantly, as already mentioned, performed mutational analysis on the majority of LQTS-related genes. The functional effect of the detected mutations was confirmed by electrophysiological methods after they were expressed in cell cultures. The study of Arnestad et al. [2] is considered as a significant milestone in the search for a genetic connection between SIDS and channelopathies. It is one of the largest studies conducted; it included 201 Norwegian SIDS cases which were strictly identified as such, according to the Nordic criteria of SIDS [33]. Gene panels were used to detect mutations in cardiac sodium and potassium channel genes. It is remarkable that this study was the first to report that approximately 9.5% of SIDS cases concealed a phenotype of LQTS. More specifically, the majority of the mutations were detected in the SCN5A gene. These mutations caused either an increased persistent sodium current or accelerated inactivation of the sodium channels. Furthermore, it has been noticed that SCN5A mutations serve as a trigger for arrhythmias during sleep [34]. This trigger, in combination with several predisposing factors (prone sleeping position, elevated sympathetic activity during REM sleep, respiratory infections), induced an enhanced cardiac electrical instability state and might subsequently lead to SIDS [35]. Mutations in the KCNQ1 gene (p.G460S) and KCNH2 gene (p.R273Q, p.R954C/p.K897T) led to a loss of function phenotype. Interestingly, certain mutations were capable of causing a fatal QT prolongation without the influence of any other triggering factor, as proven when they were expressed in laboratory animals.

Focusing on the most recent studies, in 2014, Wang et al. [18] separated another large and ethnically diverse cohort of 274 cases of sudden unexplained death in two groups, infants (141 cases) and non-infants (133 cases). Mutations responsible for LQTS were detected in 13.4% of infant cases, most frequently in the SCN5A gene. As in other broad genetic studies SIDS-related mutations were detected in genes which encode the potassium channel complex (KCNQ1, KCNH2, KCNE2), too.

Several studies focused on one single gene, rather than using gene panels. Cronk et al. [26] considered the possibility of CAV3 gene mutations resulting in SIDS. Their hypothesis was based on the observation that CAV3 mutations strongly affect the SCN5A sodium channel by inducing a persistent late sodium current thus serving as a substrate for LQTS [36]. They concluded that 2.2% of cases were due to CAV3 mutations. Tan et al. [24] analyzed another large cohort of 292 SIDS cases, submitted to the Mayo Clinic Windland Smith Rice Sudden Death Genomics Laboratory. They noticed that 1% of cases were due to variants of the SNTA1 gene, which increased peak and late sodium current and led to LQTS type 12. Cheng et al. [25] performed mutational analysis of the SCN4B gene, using the above-mentioned cohort. The variant p.S206L increased late sodium current and possibly led to LQTS type 10.

## Conclusions and discussion

The sudden death of an infant is a devastating event, which raises two major concerns for the family. First, the grief for the unexpected loss of an apparently healthy infant and second, the uncertainty for future childbirths. Subsequently, SIDS cases should be managed by a multidisciplinary team of scientists consisting of a variety of specialties such as pediatricians, pediatric cardiologists as well as adult cardiologists, pathologists, coroners, geneticists and psychiatrists. As we can infer from the tables provided, several cardiac ion channel mutations are possibly SIDS causative. However, no solid criteria based on genetic testing have been established in order to point out high risk infants. This is due to the fact that each affected infant has its unique mutational profile regarding the implicated genes. The majority of the studies conducted are based solely on post-mortem genetic analysis and due to ethical regulations the identity of the victims cannot be revealed. As a result, no family pedigree analysis can be performed nor genetic counseling provided. Moreover, no ideal screening method has been proposed, yet. In 2011, the Heart Rhythm Society (HRS) and the European Heart Rhythm Association (EHRA), published recommendations concerning the genetic testing of individuals for channelopathies and cardiomyopathies. Family genetic screening is recommended for all first degree relatives of a member affected by LQTS, BrS and CPVT but no universal neonatal screening is discussed or mentioned [37].

The usefulness of electrocardiographic (ECG) screening as a universal method for identifying the affected infants was pointed out by Schwartz et al. in 2002 [38]. To their view, a neonatal ECG is commonly unknown territory to the eyes of an adult cardiologist. Therefore, the aim of their report was to provide simple guidelines for its interpretation. The efficacy of ECG screening is addressed by two independent studies, carried out in 2000 and 2006 [39, 40]. Both focused on the LQTS, as it is the most common channelopathy among BrS, CPVT and SQTS and mainly analyzed the cost effectiveness of ECG screening. The first study of Zupancic et al. in 2000 [39], appears to have two possible weaknesses: the underestimation of the prevalence of LQTS (1/10,000) and the time point of ECG screening, which was performed early enough (day 3 of life) to confuse the normal prolongation of the QT interval during the neonatal period with the pathological one. Quaglini et al. in 2006 had more studies investigating the prevalence of LQTS at their disposal and they considered its prevalence to be 1/2500, much closer to reality [40]. Moreover, the ECG screening was performed during the 3rd or 4th week of life, a period more sensitive to identify the affected infants. Both studies suggest that universal neonatal ECG screening, despite the false negative or false positive results (which are unavoidable since the QT interval is not stable throughout the neonatal period and some infants carrying causative mutations may not have ECG findings), remains a cost-effective method.

As far as genetic screening is concerned, efficient mapping of the responsible genes could lead to the development of targeted, rapid genetic tests for the most common ones. In 2009, Redpath et al. [41] published a case report of a 22 year old man with a borderline shortening of the QT interval and proposed the use of rapid direct DNA sequencing in order to facilitate the diagnosis of the SQTS. The test yielded positive results in less than 72 h by identifying a mutation in the KCNH2 gene. Rapid genetic tests for Long QT Syndrome would be more challenging to develop since 16 genes have been found to cause LQTS, out of which more than 7 have been commonly linked to SIDS cases [42]. However, it would be naive to consider that the less common ones are not likely to contribute to SIDS. The most glaring example is represented by mutations on calmodulin, which can clearly lead to deaths that would be interpreted as SIDS [43]. Next generation whole genome sequencing (WGS) seems to achieve promising results. Rapid molecular diagnosis of LQTS at 10 days of life by WGS has been described by Priest et al. [44]. The test provided accurate readings in 8 days. In the case of deceased infants, when family genetic counseling and screening is required, molecular autopsy could be efficiently performed by WGS of DNA extracted from stored dried blood spot cards (also known as Guthrie cards, tiny blood samples obtained by pricking the infant’s heel). Breakthrough techniques allow the amplification of DNA received from such samples and are able of yielding reliable and rapid results [45]. However, parents should be aware that the detection of a mutation by molecular autopsy does not guarantee the appearance of an arrhythmogenic phenotype in the family nor justifies the exact cause of death for the infant. Last but not least, future research should aim to identify the pathogenetic mechanisms at the core of SIDS. This will enrich our knowledge and understanding of such a tragic event and help us develop more efficient preventive measures as well as improved counseling.

## Abbreviations

SIDS: sudden infant death syndrome
LQTS: long QT syndrome
BrS: Brugada syndrome
CPVT: catecholaminergic polymorphic ventricular tachycardia
SQTS: short QT syndrome
WGS: whole genome sequencing
